# Who is using take-home naloxone? An examination of supersavers

**DOI:** 10.1186/s12954-022-00647-z

**Published:** 2022-06-18

**Authors:** Desiree Eide, Philipp Lobmaier, Thomas Clausen

**Affiliations:** 1 Norwegian Centre for Addiction Research, University of Oslo, Oslo, Norway; 2 Division of Mental Health and Substance Abuse, Diakonhjemmet Hospital, Oslo, Norway

**Keywords:** Opioid, Heroin, Overdose, Harm reduction, Substance use

## Abstract

**Background:**

As the opioid overdose crisis persists and take-home naloxone (THN) programmes expand, it is important that the intervention is targeted towards those most likely to use it. We examined THN program participants to 1) describe those that return for refills, specifically those that reported multiple use (supersavers), and 2) to examine what rescuer characteristics were associated with higher rates of THN use.

**Methods:**

This study included a cohort of consenting THN recipients from June 2014–June 2021 who completed initial and refill questionnaires from a widespread program in Norway. Age, gender, number of witnessed and experienced overdoses were assessed for associations with higher reported rates of THN use. ‘Supersavers’ reported 3 or more THN uses.

**Results:**

A total of 1054 participants returned for a THN refill during the study period. Of these, 558 reported their last THN to have been used on an overdose. Supersavers (those that reported 3 or more THN uses) were younger, primarily reported current opioid use, and had witnessed higher rates of overdoses at the time of initial training when compared to non-supersavers (those that reported 0–2 THN uses).

**Conclusions:**

THN programs should continue to emphasize and prioritize THN for people actively using drugs, particularly those who have witnessed overdoses previously.

## Background

Overdoses are a significant public health issue and are responsible for an estimated 200,000 deaths globally each year [1]. Opioids are suspected to be the cause of the majority of these deaths [1], and many of these deaths can be prevented with the timely use of naloxone. Take-home naloxone (THN) programmes emerged in the 1990s in attempt to reduce opioid overdose deaths by equipping bystanders with naloxone, an opioid antagonist [2]. Since THN programmes prepare bystanders to reverse an overdose, they must reach not only those that are likely to overdose themselves, but also and primarily those likely to witness an overdose.

Peer administration of THN (often alongside overdose prevention training) has been acknowledged as a key intervention in preventing overdose deaths [3, 4]. On a macro-level, THN programmes have been found to reduce overdose mortality [5] while also being cost-effective. Economic modelling studies have shown THN to be cost-effective [6, 7], but only when distribution is targeted towards people who have a high enough risk [8].

When examining those who are at high-risk of overdosing, previous studies have shown that rescues are often performed by people who use drugs [9–11]. A study in New York found that training individuals at high-risk of witnessing an overdose resulted in frequent use of THN, with naloxone being used in 77% of the witnessed overdoses [9]. Within the opioid use disorder (OUD) treatment setting, a yearlong cohort study found that nearly 20% of the OUD patients enrolled in the study had reversed an overdose within the year [12].

As the opioid overdose crisis persists, and THN programmes expand, it is important that this intervention is targeted towards those most likely to use it. Budget constraints and resource scarcity that THN programmes commonly face mean it is important to optimize programmes [13, 14]. Also, given that this group is often hard to reach, specific and targeted outreach is essential. A more detailed description and understanding of who uses THN (a lot), i.e. ‘supersavers’, can help to guide the implementation of THN programmes to better reach those with the greatest potential of rescuing someone.

In this study, we investigated a cohort of THN programme participants and aimed to 1) describe those that return for refills, specifically those that reported multiple use of naloxone (supersavers), and 2) to examine what rescuer characteristics were associated with higher reports of THN use.

## Methods

### Design and setting

This study is a prospective cohort study based on data from consenting participants in the Norwegian THN programme from the period of June 2014 to June 2020. All individuals receiving THN were asked to complete questionnaires and provide consent to use their data for the study. Participation in the study was voluntary and did not preclude receiving THN. Because participants could receive THN without enrolling in the study, unique identification data were not captured on all participants at first distribution of kits. This reflected a decided priority in distributing naloxone over requiring complete registration and thus data collection from all participants.

The Norwegian THN programme is a widespread, government-supported public health initiative. The programme began as a pilot project in June 2014 as part of the National Overdose Prevention Strategy [15]. Details for the THN programme are described elsewhere [16]. Presently, the Norwegian THN programme is a national scheme, currently offering naloxone at 125 distribution sites across the country. Naloxone is distributed within the national overdose prevention scheme without individual prescription or cost to recipients.

Data collection consists of (ideally) an initial baseline questionnaire at the first training, followed by a refill questionnaire for all subsequent visits. A participant ID is generated using the first three letters of their last name and the first four digits of their birthdate. This ID was used to link baseline and refill questionnaires across sites. Data are collected by staff working at the distribution sites at the point of THN training [17]. Data collection began on paper in 2014 but transitioned to electronic data collection for all sites by 2018. Participation in data collection is voluntary, and individuals can receive naloxone without filling in the questionnaires (at any time point).

### Measures

The measures examined come from two separate questionnaires. The measures obtained from the baseline questionnaire include gender, age, current opioid use (current, previous, never), number of witnessed overdoses (never, once, 2–10 times, 11–20 times, more than 20 times), and the number of experienced overdoses (never, once, 2–10 times, 11–20 times, more than 20 times). From the refill questionnaires, we obtained: the rescuer’s relationship to the person who overdosed (friend, acquaintance, partner, stranger, self, child, other), the overdose location (private home, shelter, on the street/public place, car/car park, other), number of reported THN saves, and number of returns for refills. This information did not overlap, and therefore, questions asked from the initial questionnaire were not repeated at subsequent refills.

### Inclusion

Participants were included in this study if they returned one or more times for a refill and had provided data at baseline.

### Supersaver

Supersavers are defined as individuals who returned for a refill three or more times during the study period and reported that the sprays had been used on an overdose. This dichotomization of supersavers (as those used THN three or more times) and not supersavers (those that used THN zero to two times) was made due to the spread of the data and the need for sufficient amounts in each group. There were no limits to how many times someone could obtain a refill from the THN programme.

### Statistical analysis

Baseline demographic characteristics (gender, age, current opioid use status, number of witnessed overdoses, and number of experienced overdoses) are described for all participants. For witnessed overdoses, we describe the overdose location and the witnesses’ relationship to the person who overdosed. Pearson Chi-square tests were used to assess the relationship between different participant characteristics (gender, current opioid use, and number of witnessed and experienced overdoses) and reported THN use (‘not used’ against ‘used’ and ‘zero to two reports’ against ‘three or more reports’) for participants who provided baseline data and refill data. Independent samples t tests were used to compare age and reported THN use (‘not used’ against ‘used’ and ‘zero to two reports’ against ‘three or more reports’).

All analyses were completed in IBM SPSS version 27.

### Ethical approval

This study was approved by the Norwegian Data Protection Official for Research.

## Results

### Demographics

The Norwegian THN programme distributed 15,708 kits from June 2014 until June 2021. Consent was received for 10,682 (68.0%) of the kits distributed. Of these, 5226 individuals completed initial baseline questionnaires and 1054 returned at least once to pick up a new spray. These 1054 make up the subset of participants in this study.

At baseline, the majority of the returning participants were male (66.7%) with a mean age of 38.2 years (Table 1). Nearly all participants used opioids (either currently or previously) (91.8%, n = 968). Over 90% (n = 953) of returning participants had witnessed an overdose at some point prior to their first naloxone training, and 61% (n = 640) had experienced at least one overdose themselves. For all returning participants (both those that reported to have used THN and not), 21% (n = 110) and 19% (n = 200), respectively, had witnessed more than 20 overdoses.

**Table 1:** Characteristics of take-home naloxone participants at initial training (n = 1054)

Characteristics	All returning participants	
	N	%
*Gender*		
Male	696	66.0
Female	351	33.3
Missing	7	0.7
*Age (mean/SD)*	38.2	10.1
Missing	45	4.3
*Current opioid use*		
Yes	721	68.4
No	333	31.6
*Witnessed overdoses*		
Never	80	7.6
Once	53	5.0
2–10 times	419	39.8
11–20 times	281	26.7
More than 20 times	200	19.0
Missing	21	2.0
*Experienced overdoses*		
Never	160	15.2
Once	49	4.6
2–10 times	411	39.0
11–20 times	99	9.4
More than 20 times	81	7.7
Missing	254	24.1

### Take-home naloxone refills and use

There were 1054 individuals who returned for a refill during the study period. Among those who returned for a refill, the median number of refills was 2 (IQR = 3). There were 558 participants (52.9%) that reported that their THN was used at least once on an overdose, and 404 reported it had not. The 558 participants that reported using THN did so at 2216 overdose incidences (Table 2). The rescuer’s relationship to the person who overdosed was nearly always known, as ‘stranger’ made up less than a quarter of cases (11.5%, n = 255). Over half of the THN uses occurred in private homes (58.5%, n = 1296), followed by on the street/public locations (27.6%, n = 611).

**Table 2:** Overview on all reported overdose incidences when take-home naloxone was used

	N	%
Total	2216	100
*Relationship to person who overdosed*		
Friend	852	38.4
Acquaintance	586	26.4
Partner	137	6.2
Stranger	255	11.5
Self	169	7.6
Family	14	0.6
Client/patient	18	0.8
Other	15	0.7
Missing	170	7.7
*Overdose location*		
Private home	1296	58.5
Shelter	66	3.0
On the street/public place	611	27.6
Car or car park	18	0.8
Treatment or service centre	23	1.0
Other	22	1.0
Unknown	15	0.7
Missing	165	7.4

Among those that had returned for a refill, there were significant differences between those that reported their last spray to have been used on an overdose and those that had not used their last spray on an overdose (i.e. lost it, gave it away, etc.). Among refillers, those that reported to have used their THN on an overdose were younger (M = 37.5, SD = 9.9; t (924) = 1.67, *p* = 0.048), reported current opioid use (χ2 = 7.34, df = 2, *p* = 0.03), had witnessed higher rates of overdoses at the time of initial training (χ2 = 15.83, df = 4, *p* = 0.003), and had experienced higher rates of overdoses at the time of initial training (χ2 = 10.74, df = 4, *p* = 0.03). There were no significant differences between gender and reported use of THN when obtaining a refill (χ2 = 0.34, df = 1, *p* = 0.56).

### Supersavers

Participant characteristics for those who used THN on an overdose are summarized in Table 3. Of those who had used their last THN on an overdose, 70.8% (n = 395) returned 1–2 times, 22.4% (n = 125) returned 3–5 times, and 6.8% (n = 38) returned for refills 6 or more times. From the total reports of THN being used for an overdose, there were 163 participants (29.2%) that reported 3 or more uses. Supersavers (those that reported 3 or more THN uses were younger (M = 36.4, SD = 9.63; t (924) = 2.13, *p* = 0.02), primarily reported current opioid use (χ2 = 6.49, df = 1, *p* = 0.01), and had witnessed higher rates of overdoses (χ2 = 18.461, df = 4, *p* < 0.001) (Table 3). While most participants who returned for a refill had experienced at least one overdose personally, 60.4% of the supersavers had experienced 2–10, and 11.1% experienced more than 20. However, these differences between the groups of those who had used naloxone less than and more than 3 times were not found to be significant (χ2 = 8.026, df = 4, *p* = 0.09). Gender was not found to be significantly different between the supersavers and non-supersavers.

**Table 3:** Characteristics of participants who returned for a take-home naloxone refill grouped by number of reported uses (n = 1054)

Characteristics	All returning participants		Used THN 0–2 times		Used THN 3 or more times		*p*-Value^a^
	N	%	N	%	N	%	
*Gender*							0.09
Male	696	66.0	544	68.1	100	61.3	
Female	351	33.3	250	31.5	62	38.0	
Missing	7	0.7	5	0.5	1	0.6	
*Age (mean/SD)*	38.2	10.1	38.3	10.1	36.4	9.6	0.02*^b^
Missing	45	4.3	27	3.4	9	5.5	
*Current opioid use*							0.01*
Yes	721	68.4	542	67.8	127	77.9	
No	333	31.6	257	32.2	36	12.3	
*Witnessed overdoses*							0.001*
Never	80	7.6	61	7.8	6	3.8	
Once	53	5.0	45	5.6	5	3.1	
2–10 times	419	39.8	329	41.2	57	35.8	
11–20 times	281	26.7	197	24.7	65	40.9	
More than 20 times	200	19.0	154	19.3	26	16.4	
Missing	21	2.0	13	1.6	4	2.5	
*Experienced overdoses*							0.09
Never	160	15.2	126	21.1	22	15.3	
Once	49	4.6	38	6.4	5	3.5	
2–10 times	411	39.0	294	49.2	87	60.4	
11–20 times	99	9.4	81	13.6	14	9.7	
More than 20 times	81	7.7	58	9.7	16	11.1	
Missing	254	24.1	202	25.3	19	11.7	

*p =  < 0.05, THN: take-home naloxone

^a^Pearson Chi-square tests comparing participant characteristics against reported THN use rates (0–2 times, 3 or more times),

^b^Independent samples t test comparing age against reported THN use rates (0–2 times, 3 or more times)

## Discussion

We found that over 90% of returning participants had witnessed an overdose at some point prior to their first naloxone training. When examining characteristics of those that used THN and not used THN at the time of refill, those that reported to have used THN were younger, had higher rates of current opioid use, and had witnessed and experienced more overdoses at the point of their initial training. Supersavers (those who reported three or more THN uses) had similar characteristics, when compared to the non-supersaver group (reported zero to two THN uses). Supersavers were associated with higher reports of current opioid use, higher rates of witnessed overdoses at the time of their initial training, and on average were younger when compared to the non-supersaver group. For those that reported using THN, most instances were on someone that the rescuer knew. Our findings suggest that those returning for a THN refill, irrespective of how many times they have used THN, should be considered a highly relevant target group for THN distribution and outreach.

The majority of the participants in this study were people who currently or previously used opioids and had witnessed and/or experienced overdoses. Over 90% of our participants had witnessed at least one overdose at the time of their initial THN training, which is consistent (albeit on the high end) with global prevalence estimates [18]. Others have suggested targeting THN programs towards people who use drugs and that these groups are the most likely to witness and respond to an opioid overdose [9, 10]. The Norwegian THN program has targeted active drug users [16], which is supported by the characteristics of participants in this study. Individuals that returned for a THN refill (even in cases when previous THN was not used) were current opioid users, which is consistent with what others have found [10, 19].

Prior studies have found witnessing an overdose a predictor for naloxone use [10]. This is in line with our main finding, as those that reported 11–20 witnessed overdoses at the initial THN training were also those that reported the highest rates of THN use. The supersaver group made up 15% of the sample but contributed to 29% of reported THN use. This further illustrates the importance of targeting this group of active drug users, particularly as THN programs expand to other potentially relevant groups, such as police, ambulance staff, and relatives of people who use drugs.

While often deemed ‘hard-to-treat’, the participants in this study returned for THN refills, even in cases when their previous THN had not been used on an overdose. In a study of ‘hard-to-reach’ people who use drugs in Norway, poly-substance injectors who were outside of treatment had a 10 times higher mortality risk when compared to the general public [20]. This group, although outside of treatment, may be more accessible via low-threshold facilities, often where THN distribution occurs. The participants in this study not only witnessed overdoses and returned to the THN distribution facilities for refills, but also had high rates of personally experiencing overdoses. Of those that returned for a refill, over 60% had previously had an opioid overdose. This illustrates the dual importance of overdose prevention training for the participant as a rescuer, but also for personal overdose prevention education.

This study has a number of limitations. First, the data provided are self-reported and therefore subject to recall bias. The questionnaires did not include a time reference period, which would have improved the interpretation of our results. In addition, baseline data were not collected for all participants who returned for a refill, so reports of overdose reversals where baseline data were not available were not included in this study. Further, reports of refills were only collected for those that returned to a distribution site, since no active follow-up occurred. We therefore may be missing overdose events when a participant did not complete baseline data, did not return for a refill questionnaire, or did not complete a refill questionnaire. Lastly, the government supported THN program and Norway, and the access to a range of distribution sites throughout the country may limit the generalizability of our findings to different settings. Despite the limitations, this study was able to observe over 1,000 participants who returned for a THN refill and to explore associations with particular characteristics and higher reports of subsequent THN use.

In summary, we found that those who reported the highest rates of naloxone use (supersavers) were associated with reporting current opioid use and having witnessed a high number of overdoses at initial THN training. Our findings support that THN programs continue to optimize access of THN to people actively using drugs, particularly those who have witnessed overdoses previously.

## Data Availability

The datasets analysed during the current study are not publicly available due to privacy for the participants but are available from the corresponding author on reasonable request.
